# Evaluation of a pilot immunization curriculum to meet competency training needs of medical residents

**DOI:** 10.1186/s12909-020-02349-1

**Published:** 2020-11-17

**Authors:** Rebecca A. Shalansky, Margaret Wu, Shixin Cindy Shen, Colin Furness, Shaun K. Morris, Donna Reynolds, Tom Wong, Barry Pakes, Natasha Crowcroft

**Affiliations:** 1 Public Health and Preventive Medicine Residency, The University of Toronto, Toronto, Ontario Canada; 2 Department of Family and Community Medicine, The University of Toronto, Toronto, Ontario Canada; 3 Dalla Lana School of Public Health, University of Toronto, Toronto, Ontario Canada; 4 Faculty of Information, University of Toronto, Toronto, Ontario Canada; 5 Division of Infectious Diseases, Hospital for Sick Children, Toronto, Ontario Canada; 6 Centre for Global Child Health, Hospital for Sick Children, Toronto, Ontario Canada; 7 Department of Pediatrics, Faculty of Medicine, University of Toronto, Toronto, Ontario Canada; 8 Faculty of Medicine, University of Ottawa, Ottawa, Ontario Canada; 9 Centre for Vaccine Preventable Diseases, University of Toronto, Toronto, Ontario Canada; 10 Department of Laboratory Medicine and Pathobiology, University of Toronto, Toronto, Ontario Canada; 11 Institute for Clinical Evaluative Sciences, Toronto, Ontario Canada

**Keywords:** Vaccination, Immunization, Public health, Post-graduate medical education, Case-based learning

## Abstract

**Background:**

Vaccination is the most cost-effective medical intervention known to prevent morbidity and mortality. However, data are limited on the effectiveness of residency programs in delivering immunization knowledge and skills to trainees. The authors sought to describe the immunization competency needs of medical residents at the University of Toronto (UT), and to develop and evaluate a pilot immunization curriculum.

**Methods:**

Residents at the University of Toronto across nine specialties were recruited to attend a pilot immunization workshop in November 2018. Participants completed a questionnaire before and after the workshop to assess immunization knowledge and compare baseline change. Feedback was also surveyed on the workshop content and process.

Descriptive statistics were performed on the knowledge questionnaire and feedback survey. A paired sample T-test compared questionnaire answers before and after the workshop. Descriptive coding was used to identify themes from the feedback survey.

**Results:**

Twenty residents from at least six residencies completed the pre-workshop knowledge questionnaire, seventeen attended the workshop, and thirteen completed the post-workshop questionnaire. Ninety-five percent (19/20) strongly agreed that vaccine knowledge was important to their career, and they preferred case-based teaching. The proportion of the thirty-four knowledge questions answered correctly increased from 49% before the workshop to 67% afterwards, with a mean of 2.24 (CI: 1.43, 3.04) more correct answers (*P* < 0.001).

Sixteen residents completed the post-workshop feedback survey. Three themes emerged: first, they found the content specific and practical; second, they wanted more case-based learning and for the workshop to be longer; and third, they felt the content and presenters were of high quality.

**Conclusions:**

Findings from this study suggest current immunization training of UT residents does not meet their training competency requirements. The study’s workshop improved participants’ immunization knowledge. The information from this study could be used to develop residency immunization curriculum at UT and beyond.

**Supplementary Information:**

The online version contains supplementary material available at 10.1186/s12909-020-02349-1.

## Background

Vaccination is the most cost-effective medical intervention known to prevent morbidity and mortality in both individuals and populations [1]. The delivery of immunization programs in Canada varies widely. Generally, public health authorities and public health specialists are engaged in vaccine program-related decision-making, public communication, safety and disease surveillance, while front-line clinicians administer vaccines, give advice, and counsel patients [2].

One cause of the rise in vaccine hesitancy and refusal [3] is parental concerns regarding vaccine safety and the necessity of immunization [4]. Parents cite physician recommendation as an important factor in their decision to have their children vaccinated [5] and physicians are considered the most trusted source of vaccine-related information [6]. Ensuring that physicians in all relevant fields have the background vaccine knowledge and the counselling skills to engage in vaccine-hesitancy counselling and safe vaccine delivery is thus critically important to addressing the phenomenon of vaccine hesitancy. This is especially true in the province of Ontario, where family physicians and pediatricians play a key role in ensuring children and adults receive their routine immunizations, including the flu vaccine [7].

Data are limited on the effectiveness of programs in providing postgraduate medical trainees undergoing clinical training, in Canada known as medical residents, with immunization knowledge and skills. A Canadian study, the VaxEd Project, conducted with nursing, medical and pharmacy school students found that 74% of participants did not feel comfortable discussing potential vaccine-related side effects, and only 21% felt that they received adequate immunization teaching [8]. This study did not include postgraduate trainees in residency programs. A study carried out in the United States reported that 82% of pediatric residency program directors were interested in having formal vaccine safety training in their programs [9]. A 2018 study found that 96% US pediatric residents felt they would benefit from receiving more information about vaccine- preventable diseases and 73% of residents were “extremely concerned” about parental vaccine refusal [10].

Relevant Canadian residency programs, including those in family medicine, public health and preventive medicine, emergency medicine, obstetrics and gynecology, pediatrics, internal medicine, adult infectious diseases, pediatric infectious diseases and medical microbiology do include some immunization knowledge competencies in their objectives of training [11]; However, most of these are very basic, vague or insufficient for practice.

Some coordinated efforts at improving resident immunization knowledge and skills do exist, such as the Canadian Pediatric Society’s Education Program for Immunization Competencies (EPIC) program. The EPIC program is targeted at general health care professionals, and does not meet any residency program’s specific learning objectives [12].

The objectives of our study were to: 1) Describe the real and perceived immunization competency needs of post-graduate resident trainees across nine medical specialties (family medicine (FM), public health and preventive medicine (PHPM), emergency medicine (EM), obstetrics and gynecology (OBGYN), pediatrics, internal medicine (IM), adult infectious diseases (ID), pediatric ID and medical microbiology (MM)) at UT; 2) Develop a pilot immunization competency-based workshop; and 3) Provide immunization content to residents via the pilot workshop, and evaluate its impact. This study is especially relevant given the shift in the Canadian medical curriculum to competency-based medical education.

## Methods

Our study population was residents at UT in nine medical specialties with immunization listed in their training objectives: family medicine, public health and preventive medicine, emergency medicine, obstetrics and gynecology, pediatrics, internal medicine, adult infectious diseases, pediatric infectious diseases and medical microbiology to participate in an immunization education workshop. Our goal was to recruit a sample of residents from this population to pilot an immunization curriculum. Attendance and participation in the workshop was optional. The study was an observational pre/post-test study. Ethics approval was obtained by Public Health Ontario’s Ethics Review Board in October 2018.

We recruited residents via emails and flyers electronically distributed by residency program directors in the fall of 2018. After confirming attendance, an online questionnaire was sent to participating residents both to survey their experiences with immunization education in their program and to establish a baseline of their immunization knowledge. Within the questionnaire, the perceived immunization needs were reflected in the initial subjective questions, while the objective subject matter questions were used to determine knowledge deficits. The complete questionnaire is available as Supplemental Digital Appendix 1. Coded responses were used to preserve resident anonymity.

Residents then participated in a half-day immunization education workshop in November 2018. The workshop consisted of both didactic lectures given by experts in the field and interactive case-based scenarios. The initial plan was to deliver the workshop over two days, but the timing was shortened to half a day at the request of the residency program directors in order to minimize time away from clinical duties. Table 1 describes the core content of the workshop.

**Table 1:** Pilot Immunization Curriculum Delivered to Seventeen UT residents in November 2018

Session	Topic
1) Introduction	a. Clinical and public health context of vaccination
2) Vaccines and risk groups	a. The immunization program schedule – description and rationale b. Immunization of high risk populations c. Vaccine myths about aluminum, thimerosal and other ingredients d. Cold chain failure – Case study
3) Outbreaks, safety and hesitancy	a. Adverse Events Following Immunization (AEFI) b. Vaccine hesitancy c. Outbreak – Case study

Both the knowledge questionnaire and half-day curriculum were designed by a committee comprised of residency program directors and faculty, vaccine experts, and resident representatives. The curriculum design process involved regular meetings where committee members shared insights from their collective experience teaching residents, advising physician practice, and developing immunization training programs. The curriculum was then supplemented with our review of the target residencies’ competency requirements.

At the end of the half-day, a survey was distributed to collect feedback from participants. The complete survey is available as Supplemental Digital Appendix 2. The twenty-two comments from the survey were transcribed, and descriptive coding was used to identify emergent themes [13]. Descriptive statistics were calculated for the quantitative data using Microsoft Excel (version 15.33).

One week after the workshop, residents completed the original online knowledge questionnaire to assess for any changes in knowledge. Pre- and post- workshop results were paired to the individual participants using their assigned codes. Descriptive statistics were calculated for both the pre- and post- knowledge questionnaire using Microsoft Excel (version 15.33). A paired sample t-test using R (version 3.5.2) was used to examine whether the before and after responses differed in proportion and number correct for residents who completed both knowledge questionnaires. The complete study period was two months, from October to November 2018.

## Results

### Knowledge questionnaire

Using the Canadian Residency Match Service (CaRMS) website [14], we can estimate that the total number of residents in the nine targeted specialties in 2018 was approximately 697 (nine EM, 322 FM, 153 IM, ten MM, sixty OBGYN, fifty-four pediatric, ten PHPM, eight ID and two pediatric ID).

We recruited twenty residents to complete the pre-workshop questionnaire, 85% (17/20) of whom also attended the workshop, representing approximately 2.4% of the population. Seventy-six percent (13/17) of those residents who attended the workshop completed the post-workshop knowledge questionnaire; twelve of them could be paired to the pre-workshop questionnaire. Half (10/20) of the individuals completing the pre-workshop questionnaire were family medicine residents. Of those who finished the post-workshop questionnaire, no resident type was in majority. The number of years of training were evenly distributed across the pre- and post- questionnaire takers (Table 2).

**Table 2:** Knowledge Questionnaire Participation by Specialty and Training Year from Pilot Immunization Workshop for UT Residents Delivered in November 2018 *N* = 20 pre workshop, *N* = 13 post workshop

Specialty	Year	Pre	Post
FM	PGY1	7	2
	PGY2	3	2
PHPM	PGY1	1	1
	PGY4	2	2
Pediatrics	PGY4	2	2
MM	PGY2	1	0
	PGY3	1	0
GIM	PGY4	1	1
	Unknown	2	3
Total		20	13

**Fig. 1:**
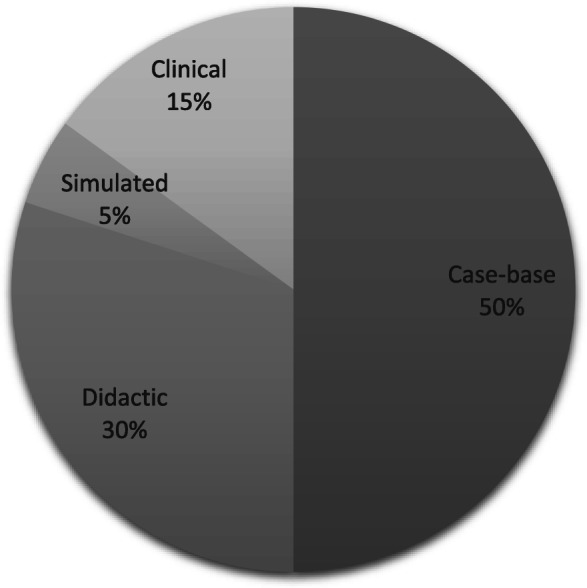
Preferred Teaching Format. Residents preferred a case-based teaching format. Most of their vaccine training to-date had been either didactic or through clinical experience

Of the twenty residents who completed the pre-workshop questionnaire, 95% (19/20) strongly agreed that vaccine knowledge was important to their future medical career while only one resident strongly agreed that they have received training sufficient to make them competent in the subject. Residents preferred a case-based teaching format, but said most of their vaccine training had been either didactic or through clinical experience (Fig. 1). Forty percent (8/20) of residents had never been taught ways to address vaccine hesitancy, half of whom were family medicine residents.

Prior to the workshop, 25% (5/20) of residents indicated that they were moderately comfortable counselling vaccine hesitant patients. After the workshop, 77% (10/13) of residents indicated that they were moderately comfortable.

Overall, the lowest baseline knowledge was in questions pertaining to adverse events following immunization, with an average of 27% correct answers for questions in this category. The greatest improvement in knowledge occurred in questions regarding public health oversight, roles and responsibilities. The average percentage of correct answers for the thirty-four knowledge questions of the survey before the workshop was 49%, whereas after the workshop the average number of correct answers was 67%. There was a mean of 2.24 (CI: 1.43–3.04) more correct answers after the workshop (*P* < 0.001). This effect was highly significant despite a decrease in effective *n* owing to participant attrition of 24% from pretest to posttest measurement.

### Feedback survey

A total of sixteen residents who attended the workshop completed the post-workshop survey. Over 90% (15/16) of the participants agreed or strongly agreed that the workshop offered new information, that the information was relevant to their practice, and that more education would be needed in the future. All residents (16/16) felt that both the didactic lectures and case studies were valuable components; 38% (6/16) of the residents strongly agreed that the lectures were valuable components while 56% (9/16) strongly agreed that the case studies were valuable components. 94% (15/16) of the residents agreed or strongly agreed that the workshop was well organized while one resident strongly disagreed with the statement (Table 3).

**Table 3:** Feedback Survey Results from Pilot Immunization Workshop for UT Residents Delivered in November 2018 *N* = 16

	Disagree/Strongly disagree	Agree/Strongly Agree
Gave me new information	**1** (6%)	**15** (94%)
Relevant to practice	**1** (6%)	**15** (94%)
Appropriate to my level of education	**3** (19%)	**13** (81%)
Other residents would benefit	**2** (12%)	**14** (88%)
I would like more education	**1** (6%)	**15** (94%)
More comfortable immunizing high risk groups	**3** (19%)	**12** (75%)
More comfortable counselling on vaccine myths	**2** (12%)	**14** (88%)
Know more about how to store vaccines	**1** (6%)	**15** (94%)
Know more about vaccination adverse events	**1** (6%)	**15** (94%)
More comfortable counselling on vaccine hesitancy	**0**	**16** (100%)
Know more about what to do in an outbreak	**1** (6%)	**15** (94%)
The lectures were a valuable component	**0**	**16** (100%)
The case studies were a valuable component	**0**	**16** (100%)
I felt encouraged to participate	**3** (19%)	**13** (81%)
Well organized	**1** (6%)	**15** (94%)
A good use of my time	**3** (19%)	**13** (81%)
Met my expectations	**3** (19%)	**13** (81%)

All 16 participants who completed the post-workshop survey also gave qualitative feedback after the workshop. Participants’ feedback clustered into three emergent themes: 1) They found the content specific and practical; 2) They wanted more case-based learning and for the workshop to be longer; 3) They felt the content and presenters were of high quality.

#### Theme 1: Specific and practical content was helpful, and more was wanted

Participants appreciated the practical advice from the workshop, which they could apply to vaccine delivery and counselling.

*“[the workshop provided] practical tips for vaccine hesitancy”.*

Participants were eager to learn not only general principles, but also specific language and methods that experts use to counsel patients and their families. For example, one participant found that comparing the probability of a rare vaccine side effect to that of being “struck by lightning” helpful in demonstrating the low risk to patients.

*“More specifics on risks of vaccines and comparisons (loved ‘struck by lightning’)”.*

#### Theme 2: More case-based learning and more time in general were wanted

To deliver theoretical and practical content, the workshop was delivered as both didactic presentations and case studies. Most of the participants preferred the case-based learning as they felt that it was more interactive and engaging.

*“Small group case studies were fantastic! Easier to ask questions/more engaging”.*

Nevertheless, participants felt that more time was needed for the didactic components of the workshop. The field of immunization is very broad, and the participants were overwhelmed by the volume of knowledge included in a half-day workshop. Participants wanted more time for the presentations and time to ask relevant questions afterwards.

*“[the workshop needed] longer and more time per speaker”.*

#### Theme 3: The content and presenters were of high quality

Participants felt that the workshop delivered relevant immunization knowledge that they could apply to their practice and residency. They could sense that the speakers at the workshop were experts in the field who offered diverse perspectives and experiences in vaccine delivery and counselling.

*“Fantastic and very relevant workshop for clinicians”.*

Overall, residents felt that the workshop was a valuable learning experience that they would recommend as being a regular part of their curriculum.

*“Would be very beneficial for residents and fellows to have this type of workshop included in the curriculum.”*

## Discussion

Immunization knowledge and skills are critically important for physicians, across many specialties, who are involved in the delivery of vaccines or vaccine information. Recent events, including increased vaccine hesitancy, declining rates of immunization, and outbreaks of vaccine preventable diseases heighten this need.

This study highlighted the gaps in immunization education in UT residency programs both in baseline core immunization knowledge as well as in vaccine counselling. We suggested an approach in the delivery of a curriculum to improve both competencies. The residents surveyed agreed that immunization is an important topic to be covered during their residency education; however, the majority did not strongly believe that they have achieved competence. While most immunization information is currently delivered didactically, the qualitative feedback indicated that residents preferred a case-based approach.

Overall, the pilot workshop was effective in improving resident’s short-term immunization knowledge and vaccine hesitancy counselling confidence. It would be valuable to determine the long-term impact of the workshop by re-surveying participating residents after a period of time, and after residency. This remains a possibility as a future direction of the study. We also plan to build on this pilot study to and offer this workshop regularly to reach more resident participants. Another future direction is to evaluate the immunization curriculum of other residency programs, as we hypothesize that a similar knowledge gap could exist in other Canadian universities and internationally. With the information gathered from this project, we plan to advocate for a stronger immunization curriculum at UT, and possibly to other programs across Canada. The workshop potentially provides a template that residency programs could incorporate into their curriculum, tailoring it to their specific program objectives.

Limitations of this study include its observational study design, making it difficult to account for confounding, controls, and causality, including test effects. We also recognize the small number of participants, as well as the lack of long term follow up. The principle recruitment challenge was the difficulty of residents being relieved of their clinical duties to participate. There was significant resident interest in having more than one half-day allotted; However, residents serve a critical role in providing patient care in the Ontario healthcare system, and removing a large group of residents from clinical duties, even for half a day, can cause significant disruption. It would have been helpful to know the number of residents who were interested in attending, but were not able to due to competing priorities. Ideally, an immunization curriculum would be an integrated and mandatory part of residency training. Furthermore, as residents volunteered for the workshop, self-selection bias may also have influenced the results. It is unknown whether residents with more, or less baseline immunization knowledge would have been keener to participate. Furthermore, having a higher proportion of FM compared to other specialty residents could have skewed the results in either direction.

Though not an issue at the time of the study’s design, the current coronavirus disease 2019 (COVID-19) pandemic highlights the significance of resident immunization competencies [15]. With the development of a COVID-19 [15] vaccine upcoming, now, more than ever, physicians will need to be comfortable counseling patients, and likely will be involved in vaccine administration.

## Conclusion

Despite immunization being a part of Canadian residency program objectives, current training of residents in this subject at UT does not appear to adequately meet resident’s competency training needs. The pilot curriculum developed by this study was successful in increasing resident immunization knowledge and could be used to develop and inform further enhancement of immunization competency training among resident physicians at UT and beyond.

## Supplementary Information

**Additional file 1.**

## Abbreviations

AEFI: Adverse Events Following Immunization
CaRMS: Canadian Residency Match Service
COVID-19: Coronavirus Disease 2019
EM: Emergency Medicine
EPIC: Education Program for Immunization Competencies
FM: Family Medicine
GIM: General Internal Medicine
ID: Infectious Disease
MM: Medical Microbiology
OBGYN: Obstetrics and Gynecology
PGY: Post Graduate Year
PHPM: Public Health and Preventive Medicine
UT: University of Toronto
