# Supplementary material for: Evaluation of a pilot immunization curriculum to meet competency training needs of medical residents
Source: BMC Med Educ. 2020 Nov 17;20:442. doi: 10.1186/s12909-020-02349-1 (PMC7671185; doi:10.1186/s12909-020-02349-1)
Supplement: Supplementary file 1 — Additional file 1. [file 12909_2020_2349_MOESM1_ESM.docx]

Evaluation of a Pilot Immunization Curriculum to Meet Competency Training Needs of University of Toronto Medical Residents

**Supplemental Digital Appendices**

**Supplemental Digital Appendix 1: Pre and Post Workshop Knowledge Questionnaire**

1. What year of training are you currently in:

__ PGY1

__ PGY2

__ PGY3

__ PGY4

__ PGY5

__ PGY6+

2. Which residency program are you in:

a. Family Medicine

b. Public Health and Preventive Medicine

c. Pediatrics

d. Obstetrics and Gynecology

e. Infectious Disease

f. Microbiology

g. Emergency Medicine

f. Other, please specify: ____

3. I think vaccine/immunization knowledge is important for my future medical career

a. Strongly agree

b. Agree

c. Disagree

d. Strongly disagree

4. I received competent teaching about vaccines/immunizations during my medical training thus far

a. Strongly agree

b. Agree

c. Disagree

d. Strongly disagree

5. The training I have received in vaccines/immunizations is from (select all that apply)

a. medical school formal teaching

b. medical school informal teaching

c. residency program formal teaching

d. residency program informal teaching

e. Other, please specify:

6. To date, my education regarding vaccines/immunizations in residency was (select all that apply)

__ case-based learning

__ didactic lecture

__ simulated patient

__ during supervised clinical practice

__ other, please specify

7. My preferred method of vaccine/immunization education would be (please rank)

__ case-based learning

__ didactic lecture

__ simulated patient

__ during supervised clinical practice

8. How comfortable are you counselling vaccine hesitant patients or families? Select one.

a. Not at all

b. Slightly

c. Somewhat

d. Moderately

e. Extremely

9. Have you been taught about ways to address vaccine hesitancy during residency training?

__ Yes

__ No

10. During what year(s) of training were you taught ways to address vaccine hesitancy? (select all that apply)

__ PGY1

__ PGY2

__ PGY3

__ PGY4

__ PGY5

__ PGY6+

11. What was the teaching format? (select all that apply)

__ case-based learning

__ didactic lecture

__ simulated patient

__ during supervised clinical practice

__ others, please specify

12. Approximately how many hours of teaching have been allotted to teaching ways to address vaccine hesitancy over your residency training (so far)? ______

13. Was your training in ways to address vaccine hesitancy evaluated by your educators?

__ Yes

__ No

14. Which of the following is true:

a. The Tetanus Diphtheria Pertussis vaccine is not safe to administer to a breastfeeding mother.

**b. Giving the Tdap Vaccine to pregnant women offers protection to her newborn.**

c. The Tdap vaccine should ideally be given in the first trimester of pregnancy.

d. The Tdap cannot be administered with Rhogam.

15. In immunocompetent patients, varicella vaccine can prevent chicken pox or reduce the severity of the disease if given within 3-5 days of exposure.

**1. True**

2. False

3. Don’t know

16. What is the maximum number of vaccines that can be given at any one time?

a. 4

b. 8

c. Depends on the vaccines

**d. There is no limit.**

e. Don’t know

17. Before what age should the first dose of rotavirus vaccine be given?

a. before 10 weeks

**b. before 15 weeks**

c. before 20 weeks

d. It can be given at any age

e. Don’t know

18. Which organism is currently not included in Ontario publicly funded routine vaccination schedule?

a) Varicella

b) Rotavirus

c) Pneumococcus

**d) Hepatitis A**

e) Don’t know

19. Which of the following vaccine-preventable diseases are reported to public health in Ontario? (select all that apply)

**a) Varicella**

b) Rotavirus

c) Pneumococcal, non-invasive

**d) Hepatitis A**

e) Don’t know

20. The minimum interval recommended between doses of the same live vaccine is:

a. 1 week

**b. 4 weeks**

c. 2 months

d. No minimum interval needed

e. Don’t know

21. Which of the following diseases can never be eradicated worldwide?

**a. tetanus**

b. polio

c. measles

d. Don’t know

22. How can passive immunity be acquired? (check all that apply)

**a. Maternal transfer of antibodies**

**b. Injection of immunoglobulin**

c. Through having a disease

d. By having a vaccine

e. Don’t know

23. Which one of the following vaccines may be live?

a. *N. meningitidis* type B

b. Hepatitis B

c. Tetanus

**d. Shingles**

e. Don’t know

24. Which one of the following vaccines may be inactivated/subunit?

a. MMR

**b. Injected influenza vaccine**

c. Rotavirus

d. Varicella

e. Don’t know

25. Local reactions to inactivated/subunit vaccines most commonly begin:

a. Immediately

**b. Within 24 to 48 hours**

c. After 2 to 3 days

d. One week after vaccination

e. Don’t know

26. Reactions after live vaccines usually:

a. Increase in frequency with each dose of vaccine

**b. Decrease in frequency with each dose of vaccine**

c. Do not change in frequency with increasing doses of vaccine

d. Don’t know

27. What is the purpose of adjuvants in vaccines?

**a. They enhance the immune response to the vaccine**

b. They reduce the risk of local reactions to vaccines

c. They inactivate the vaccine when it reaches its expiry date

d. They improve the taste of oral vaccines

e. Don’t know

28. Aluminum is not present in which vaccine in Ontario?

a. Hep B Vaccine

b. Tdap

c. Pneumococcal

**d. MMR**

e. Don’t know

29. Thimerosal is metabolized into ethyl mercury in the body. Thimerosal may be present in which vaccines available in Canada?

**a. Some influenza vaccines**

b. Some shingles vaccines

c. Some pneumococcal vaccines

d. Some measles vaccines

e. All of the above

f. Don’t know

30. Which of the following is not true about thimerosal?

a. It prevents the growth of bacteria in vaccines.

b. It stays in the body for a long time.

c. It contains methylmercury.

d. **It is in all influenza vaccines.**

e. Don’t know

31. If a family with a young baby were intending to travel before their child’s first immunizations were due, the youngest age at which primary immunizations are generally recommended to start is:

a. 4 weeks of age

b. 5 weeks of age

**c. 6 weeks of age**

d. 7 weeks of age

e. Don’t know

32. Which of the following medications may be a contraindication to the administration of live vaccines?

a. Antihistamines

b. Anti-inflammatories

**c. Corticosteroids**

d. Statins

e. Don’t know

33. Immunosuppressed patients should generally not receive live vaccines because

a. They will not be able to make an immune response to them.

**b. They may cause severe disease.**

c. They are likely to already have sufficient levels of antibodies.

d. They are likely to experience more local reactions.

e. Don’t know

34. Which of the following is true about Pneumococcal vaccines (Pneu-C-13 (Prevnar) and Pneu-C-23 (Pneumovax))?

**a. Pneu-C-13 is indicated for those 50 years and above with an immunosuppressive condition.**

b. Pneu-C-23 is recommended for children under two years of age.

c. If an adult over 65 years has received Pneu-C-23 they should not be vaccinated with Pneu-C-13.

d. Pneu-C-23 and Pneu-C-13 should be administered at the same time

e. Don’t know

35. As a clinician what should you do about a suspected case of measles? (select all that apply)

a) Report to local public health unit after lab confirmation

**b) Report to local public health unit** **immediately**

c) Order serology only

**d) Instruct case to self-isolate until contacted by public health for next steps**

**e)** Report to Public Health Ontario immediately

f) Don’t know

36. Which of the following should be done when managing a contact of measles? (select all that apply)

**a) Provide MMR vaccine to susceptible immunocompetent individuals aged 6 months or older within 72 hours of exposure**

b) Provide MMR vaccine to susceptible immunocompetent individuals aged 12 months or older within 72 hours of exposure

**c) Provide immunoglobulin (Ig) to all children below 6 months of age as soon as possible within 6 days after exposure**

**d) Immunocompromised and pregnant individuals should receive immunoglobulin.**

**e) Ensure that anyone who is eligible but unvaccinated is offered MMR vaccine**

f) Don’t know

37. Which of the following is/are true if a child has received a dose of MMR vaccine before the age of 12 months? (select all that apply)

a) They need only one further dose of MMR given at the same time as the pre-school booster immunizations

**b) They need two further doses of MMR vaccine given at 12-13 months and at the same time as the pre-school boosters**

**c) The response to a dose given before 12 months may be suboptimal due to persistence of maternal antibodies in the baby**

d) This may increase the risk of an adverse reaction to any subsequent dose

e) Check measles antibodies before deciding to give a further dose

f) Don’t know

38. What are the criteria for reporting AEFIs? (select all that apply)

a**. No other clear alternative cause at time of reporting**

b**. Temporal association with vaccine administration**

c. Causal relationship

d. Serious adverse events that are life threatening or result in hospitalization or permanent disability

e. Unexpected and not included in product monograph

f. Don’t know

39. Which of the following are correct if you are a clinician reporting AEFIs in Ontario? (select all that apply)

a. Report to Health Canada

**b. Report to Local public health unit**

c. Report to Public Health Ontario (PHO)

d. Report to National Advisory Committee on Immunization

**e. Vaccine recipients and their caregivers can submit AEFI reports.**

**f** **No need to report injection site reactions that last less than four days.**

g. Don’t know

40. What is the recommended temperature range for the storage of most routinely used vaccines?

a. 0⁰C to +8⁰C

b. +2⁰C to +10⁰C

**c. +2^o^C to +8^o^C**

d. 0⁰C to +10⁰C

e. -5⁰C to -8⁰C

f. Don’t know

41. How often should the fridge temperature be read and recorded?

a. Every day before clinic begins

b. Each evening before the clinic closes

**c. Ideally twice a day at the same time**

d. Every Monday morning and Friday evening

e. Don’t know

42. Which of the following is true about vaccine distribution?

**a. Orders should be submitted to Ontario Government Pharmaceutical and Medical Supply Service (OGPMSS).**

b. Vaccines can be delivered by any courier or mail services.

c. Vaccines need to stay at room temperature for 4 hours after being delivered.

d. Vaccine ordering schedules are once every 2 months or 4 months.

e. Don’t know

43. The following are recommended interventions to reduce pain associated with vaccination among infants. (select True or False or Don’t know for each intervention)

| **True** | False | Don’t know | Aspiration when administering intramuscular vaccines |
| --- | --- | --- | --- |
| True | **False** |  | Rub the skin after vaccine injection |
| **True** | False |  | Breastfeeding during vaccine injections |
| True | **False** |  | Give the MOST painful vaccine first if multiple vaccines are injected |
| True | **False** |  | Use acetaminophen (Tylenol, Tempra) or ibuprofen (Advil, Motrin) |

**Supplemental Digital Appendix 2: Post Workshop Feedback Survey**

|  | Strongly Disagree | Disagree | Agree | Strongly Agree |
| --- | --- | --- | --- | --- |
| The content of the workshop was new information for me |  |  |  |  |
| The content of the workshop is relevant to practice in my specialty |  |  |  |  |
| The workshop was appropriate to my level of education |  |  |  |  |
| I think that other residents in my specialty would benefit from this workshop |  |  |  |  |
| After this workshop, I would like more education on immunizations/ vaccinations |  |  |  |  |
| After this workshop I would feel more comfortable immunizing high risk groups |  |  |  |  |
| After this workshop I would feel more comfortable counselling on vaccine myths |  |  |  |  |
| After this workshop I know more about how to store vaccines |  |  |  |  |
| After the workshop I know more about adverse events following vaccination |  |  |  |  |
| After the workshop I would feel more comfortable counselling on vaccine hesitancy |  |  |  |  |
| After this workshop I know more about what to do in an outbreak |  |  |  |  |
| The lectures were a valuable component of the workshop. |  |  |  |  |
| The case studies were a valuable component of the workshop. |  |  |  |  |
| I felt encouraged to participate in the discussions and activities of this workshop. |  |  |  |  |
| The workshop was well organized |  |  |  |  |
| Participating in this workshop was a good use of my time |  |  |  |  |
| Overall, this workshop met my expectations |  |  |  |  |

Please provide any additional comments on aspects of the workshop you have rated above:

Please provide any specific comments about the workshop presenters:

Is there anything about the workshop that you would very much like to see changed?

Is there anything about the workshop you would very much like to see kept the same?

Any additional comments?
